# Multiple and multidrug resistance in *Botrytis cinerea*: molecular mechanisms of MLR/MDR strains in Greece and effects of co-existence of different resistance mechanisms on fungicide sensitivity

**DOI:** 10.3389/fpls.2023.1273193

**Published:** 2023-10-05

**Authors:** Georgios Sofianos, Anastasios Samaras, Georgios Karaoglanidis

**Affiliations:** Laboratory of Plant Pathology, Faculty of Agriculture, Forestry and Natural Environment, Aristotle University of Thessaloniki, Thessaloniki, Greece

**Keywords:** ABC transporters, gray mold, fungicide resistance, MFS transporters, *mrr1* gene

## Abstract

*Botrytis cinerea* is a high-risk pathogen for fungicide resistance development. Within the fungal populations, strains have developed multiple mutations in different target genes leading to multiple resistance (MLR) or mutations associated with overexpression of efflux transporters leading to multidrug resistance (MDR). These types of resistance are a major threat, and their successful management is a major challenge. The current study was initiated to a) determine frequencies of MLR/MDR strains in populations originating from several crops, b) identify the types of MDR that occur in Greece, and c) determine interactions between MLR and MDR at the level of sensitivity to botryticides. The frequencies of MLR/MDR phenotypes were determined in 515 isolates subjected to bioassays using discriminatory concentrations of thiophanate-methyl, iprodione, cyprodinil, fenhexamid, boscalid, fluopyram, fludioxonil, pyraclostrobin, and tolnaftate. Interestingly, 7.8% and 31.3% of isolates from strawberry and rootstock seedlings were resistant to every single fungicide class, while MDR phenotypes from strawberries, rootstocks, and tomatoes accounted for 26%, 87%, and 13.4%, respectively. The MLR and MDR isolates were further molecularly analyzed regarding genes *erg27*, *sdhB*, *Bcpos5*, and *Mrr1*, responsible for resistance to fenhexamid, boscalid and fluopyram, cyprodinil, and MDR, respectively. The different mutations’ presence was determined along with a new mutation in *Mrr1* leading to MDR. MDR isolates were characterized as MDR1 or MDR1h based on the presence of a 3-bp deletion in *Mrr1*. MDR1h was predominant in isolates from rootstocks and MDR1 from tomatoes and strawberries, whereas the most frequent target-site mutations were F412S (*erg27*), H272R (*sdhB*), and L412F (*Bcpos5*). To determine whether the accumulation of target-site mutations along with MDR mutations exhibits an additive effect concerning fungicide resistance, the sensitivity of isolates possessing the predominant target-site mutations was calculated in both the presence and the absence of MDR-associated mutations. EC_50_ in cyprodinil and boscalid increased to about twofold in the presence of MDR mutations, while there was no difference for fenhexamid. In conclusion, MLR/MDR frequencies are notably high in heavily treated crops in Greece, and the combination of MLR and MDR mutations leads to even higher fungicide resistance levels, highlighting the importance of resistance management.

## 1 Introduction

Gray mold caused by *Botrytis cinerea* Pers.Fr (teleomorph *Botryotinia fuckeliana* (de Bary) Whetzel) is one of the most destructive diseases of high-value crops throughout the world. The pathogen can infect more than 1,400 plant species, among which major agricultural crops are included such as grapes, greenhouse-grown vegetables, ornamental plants, and stone, pome, and small berry fruits. It causes mostly pre- or postharvest fruit decays and, in addition, leaf blights or leaf spots, stem cankers, flower rots, and damping off of seedlings (Molly and Grant-Downton, 2015; Shaw et al., 2016). Based on its extremely broad host range and its scientific and economic significance, *B. cinerea* is ranked as the second most important plant pathogenic fungus (Dean et al., 2012). Currently, efforts to develop new disease management methods that are highly effective and environmentally friendly are intensive and include RNA interference, biological control, or plant resistance inducers (Roca-Couso et al., 2021; Samaras et al., 2021; Spada et al., 2023). However, none of these methods can provide control efficacy similar to that provided by fungicides, and thus, chemical control remains the major gray mold management method under conditions favorable for the development of the disease. A wide array of fungicides that belong to several target-site- specific chemical classes, such as the anilinopyrimidines (Aps; FRAC 9), sterol biosynthesis inhibitors class III and SBIs class III (FRAC 17), phenylpyrroles (PPs; FRAC 12), quinone outside inhibitors (QoIs; FRAC 11), succinate dehydrogenase inhibitors (SDHIs; FRAC 7), benzimidazoles (MBCs; FRAC 1), and dicarboximides (DCs; FRAC 2), are available against the pathogen (Fillinger and Walker, 2016).

However, successful control of gray mold with the use of fungicides is hampered by resistance development, one of the major restricting factors in the success of chemical control because of reduction or even complete loss of control efficacy that may lead to failures in disease management. Such problems have been faced in the past in *B. cinerea* populations that have developed resistance worldwide to all site-specific fungicides, associated with target site modifications due to mutations’ presence in genes encoding the target site of the fungicides (Brent and Hollomon, 2007; Hahn, 2014; Fernandez-Ortuno et al., 2017; Fillinger and Walker, 2016; Shao et al., 2021). More recent studies have shown that in pathogen populations throughout the world, strains have been selected possessing more than one target-site alternation conferring resistance to the respective fungicides and leading to multiple resistance (MLR) to fungicides (de Miccolis Angelini et al., 2014; Rupp et al., 2017; Naegele et al., 2022).

In addition to target site modifications, more mechanisms have been associated with fungicide resistance development in *B. cinerea*. Among them, overexpression of efflux transporters located in the plasma membranes plays a crucial role and leads to resistance development in several chemically and structurally unrelated compounds (multidrug resistance (MDR)). Two major families of efflux transporters have been recognized and associated with resistance to fungicides in fungal species: ABC transporters (ATP-binding Cassette superfamily) and MFS transporters (Major Facilitator Superfamily transporters) (de Waard et al., 2006). The evidence for MDR due to increased fungicide efflux in plant pathogenic filamentous fungi is relatively limited. So far, field strains overexpressing ABC and MFS transporters have been described in *B. cinerea*, *Zymoseptoria tritici*, *Penicillium digitatum*, and *Penicillium expansum* (Kretschmer et al., 2009; Omrane et al., 2015; Samaras et al., 2020). In *B. cinerea*, four different types of MDR strains have been recognized (MDR1, MDR1h, MDR2, and MDR3) differing in both their spectrum and level of resistance to fungicides and linked with overexpression of *atrB*- and *mfsM2*- encoded efflux transporters (Kretschmer et al., 2009; Mernke et al., 2011; Leroch et al., 2013). In addition to differences in fungicide sensitivity levels, the various MDR types may also differ in their fitness level, although further investigations are required to clarify this issue (Kretschmer et al., 2009; Chen et al., 2016).

In Greece, *B. cinerea* has developed resistance to almost all the target- site-specific botryticides that have been used for its control on several crops (Bardas et al., 2008; Samuel et al., 2011; Veloukas et al., 2011; Chatzidimopoulos et al., 2013). Recently, it became evident that in fungal populations originating from major crops heavily treated with botryticides, subpopulations with multiple resistance have been built up, but information related to their frequencies remains scarce (Bardas et al., 2010; Konstantinou et al., 2015; Makris et al., 2022). However, nothing is known related to the presence of fungal strains possessing resistance mechanisms that are associated with MDR.

The development of resistance to fungicides by *B. cinerea* is a major threat to modern agriculture, and its successful management is a major challenge. With varying levels of resistance to the main fungicides now present in the fungal populations throughout the world, coupled with the expected decrease in the number of fungicide active ingredients available to European farmers as a result of the European Green Deal, a better understanding of how resistance develops and evolves becomes increasingly important. Furthermore, MLR or MDR is hard to manage with the most common tactics of fungicide resistance management that include alternations or use of fungicide mixtures. Therefore, knowledge of the composition of *B. cinerea* populations in terms of MLR or MDR frequencies is a prerequisite for the development and application of resistance management strategies. In addition, although the effect of MDR is considered to be low for plant pathogens since it is in general associated with low/moderate resistance levels, the effect on fungal sensitivity of its combination with target-site modifications remains largely unknown. Thus, this study was initiated aiming to i) determine frequencies of MLR and MDR in pathogen populations originating from different hosts, ii) identify mutations associated with either target site modifications or increased efflux activity of membrane transporters, and iii) determine the effect on the levels of resistance to fungicides of the simultaneous presence in the same strains of different resistance mechanisms. A preliminary report on parts of this study has already been presented at the 1st Joint Botrytis-Sclerotinia Symposium 2022 (Sofianos et al., 2022).

## 2 Materials and methods

### 2.1 Sampling of isolates

**Figure 1:**
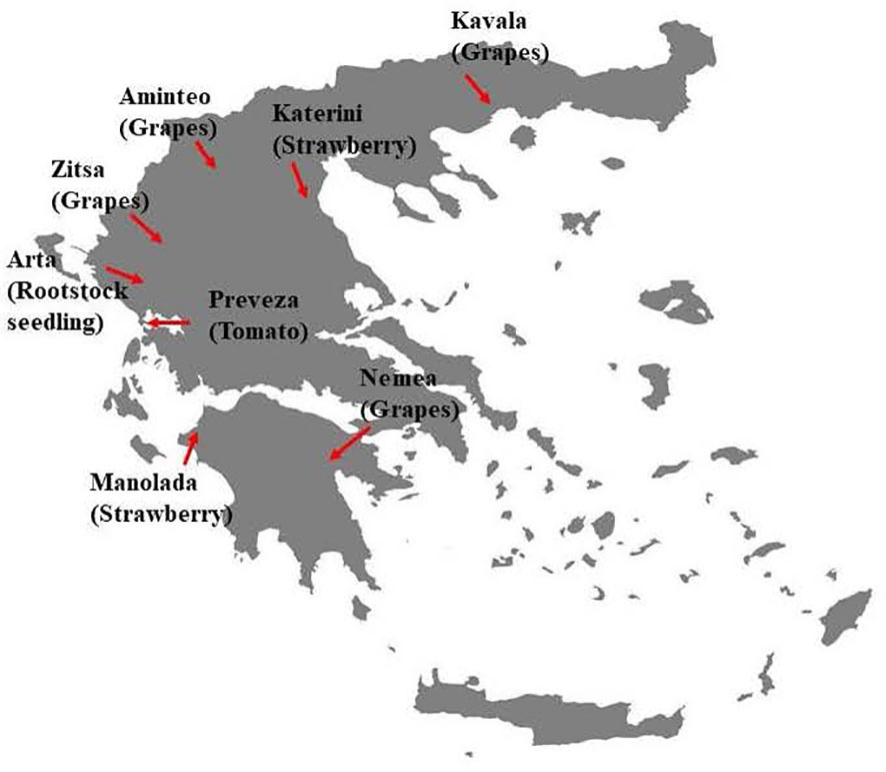
Map of Greece showing the sampling areas of *Botrytis cinerea* isolates used in the current study.

Pathogen isolates were collected during 2019 and 2020 from infected fruit of tomatoes, grapes, and strawberries showing rot symptoms and infected stone-fruit rootstock seedlings showing damping-off symptoms. Sampling was conducted in several regions, all located around mainland Greece (**Figure 1**). The samples were collected from plant organs with abundant sporulation on their rotten surface. Conidia were harvested from the sporulating lesions using sterile cotton swabs, followed by their transfer to acidified potato dextrose agar (PDA) medium (lactic acid at 0.5 ml/liter [vol/vol]) to suppress bacterial contamination. After 20 h of incubation at 22°C, the germinated spores were observed under a dissecting microscope, and from each sample, a single germinated spore was transferred to fresh PDA plates. The single-spore cultures were incubated at 20°C for 7 days and then conserved at 4°C for later use. In total, 515 isolates were obtained and used in this study. Details concerning the number of isolates per region and host are provided in **Table 1**.

**Table 1:** Number of *Botrytis cinerea* isolates collected from several hosts and several regions of Greece during 2019 and 2020.

Region	Host	Number of isolates
Preveza	Tomatoes	126
Katerini	Strawberries	60
Manolada	Strawberries	82
Arta	Stone-fruit rootstock seedlings	67
Kavala	Grapes	38
Nemea	Grapes	94
Zitsa	Grapes	44
Amynteo	Grapes	4

### 2.2 Fungicide sensitivity assays and phenotypic characterization

To determine the fungicide resistance profile of the collected isolates, previously developed protocols and determined discriminatory concentrations of fungicides were used (Leroch et al., 2011; Weber, 2011; Testempasis et al., 2020). Fungicides tested were as follows: the MBC thiophanate-methyl (Neotopsin 70WG, K+N Efthimiadis, Greece), the DC iprodione (Rovral 50SC, BASF, Greece), the AP cyprodinil (Chorus 50WG, Syngenta, Greece), the PP fludioxonil (Geoxe 50WG, Syngenta, Greece), the QoI pyraclostrobin (Comet 20EC, BASF, Greece), the SBI class III fenhexamid (Teldor 50WG, Bayer CropScience, Greece), the SDHI boscalid (Cantus 50WG, BASF, Greece), fluopyram (Luna Privilege 50 SC, Bayer CropScience, Greece) and tolnaftate (Sigma Co., St. Louis, MO, USA). The assays were performed on a solid agar medium containing the following discriminatory concentrations: thiophanate-methyl at 5 mg/L, fenhexamid at 3 mg/L, iprodione at 7.5 mg/L, boscalid at 7.5 mg/L, cyprodinil at 8 mg/L, fludioxonil at 1 mg/L, fluopyram at 2 mg/L, and pyraclostrobin at 25 mg/L (with 100 mg/L of salicylic hydroxamic acid; SHAM, Sigma Co., St. Louis, MO, USA) (Weber and Hahn, 2011; Leroch et al., 2013; Testempasis et al., 2020; Samaras et al., 2021).

To determine the resistance profile of the collected isolates to thiophanate-methyl, iprodione, pyraclostrobin, fludioxonil, and fenhexamid, conidial suspension (2 × 10^5^ spores/ml) aliquots (20 μl) of each isolate were prepared and then applied to hydroxyapatite (HA) medium (4 g of yeast extract, 4 g of glucose, 10 g of malt extract, and 15 g of agar per liter of medium), amended with the respective fungicide discriminatory concentrations. Likewise, the sensitivity to cyprodinil was determined with the transfer of the conidial suspension to Gamborg B5 medium (3 g of Gamborg, 1.36 g of KH_2_PO_4_, 9.9 g of glucose, and 15 g of agar per liter of medium, pH 5.5), amended with cyprodinil at the respective discriminatory concentration. After 72 h of incubation at 20°C in the dark, mycelial growth was evaluated. Isolates showing clear, visible mycelial growth were classified as resistant, while the absence of such growth characterized sensitive ones.

To determine the resistance profile of isolates to boscalid and fluopyram, conidial suspensions (40 μl) were prepared and spread onto yeast-bacto-acetate-agar (YBA) (10 g of yeast extract, 10 g of Bacto peptone, 20 g of sodium acetate, and 15 g of agar per liter of medium), amended with the discriminatory concentrations of boscalid or fluopyram. After 20 h of incubation at 20°C in the dark, the conidia were examined microscopically for germination. Isolates were classified as either sensitive based on the absence of conidial germination or resistant based on the presence of conidial germination. Conidia that were considered resistant possessed a germ tube at least two times longer than the conidium length.

To determine whether any of the isolates is MDR, all the isolates were subjected to conidial germination assay on sucrose agar (SA) plates amended with tolnaftate at the concentration of 5 μg/ml (Chapeland et al., 1999). The plates were incubated at 20°C in the dark, and the isolates with germinated conidia were considered tolnaftate-resistant. A conidium was considered germinated when its germ tube had a length at least two times longer than the conidium length.

### 2.3 DNA extraction

DNA was extracted from fungal cultures grown in Potato Dextrose Broth (PDB) (Sigma-Aldrich, St. Louis, MO, USA) for 3 days at 22°C as previously described by Konstantinou et al. (2015), using the QIA Puregene Core Kit A (Qiagen GmbH, Hilden, Germany). DNA concentration and purity were measured using a P330 nanophotometer (Implen GmbH, Munich, Germany).

### 2.4 Identification of target-site mutations in *sdhB*, *erg27*, and *Bcpos5* genes

In isolates that were found to be phenotypically resistant to boscalid, fenhexamid, and cyprodinil, site modifications in the respective target genes *sdh*, *erg27*, and *BcPos5* were identified with direct sequencing. The primer pair IpBcBeg/IpBcEnd, and primers SdhC1 to SdhC5 and SdhD1-D2 developed by Leroux et al. (2010) were used to amplify *sdhB*, *sdhC*, and *sdhD* subunits in all the isolates found to be resistant to the SDHIs. Similarly, *erg27* gene of all the fenhexamid-resistant isolates was amplified using the primer pair erg27Beg/erg27End (Fillinger et al., 2008). To identify mutations conferring resistance to APs, target gene *BcPos5* of the resistant isolates was amplified (Mosbach et al., 2017). For this purpose, the primer pair BcposF/BcposR2 was designed for both amplification of the product and Sanger sequencing, yielding a 1,398-bp product, while the primer pair BcposF2/BcposR was used only for the sequencing process. The PCR products were purified with Monarch^®^ PCR & DNA Cleanup Kit (NEB) according to the manufacturer’s protocol and then subjected to Sanger sequencing for the identification of the resistance-conferring mutations in *sdh*, *erg27*, and *BcPos5* genes. The primers used for target gene amplification are listed in **Supplementary Table 1**.

### 2.5 Genotypic characterization of MDR isolates

To molecularly characterize the different MDR types, routine identification of MDR1h isolates was conducted by amplifying an *mrr1* gene fragment using the primer pair *Mrr1*_spez_F/*Mrr1*-Pira, followed by digestion with HpyCH4V (Leroch et al., 2013). To test for the presence of MDR2 and MDR3 types, the *mfsM2* upstream region was amplified with the primer mfsM2-pfor/mfsM2-prev (Kretschmer et al., 2009). In order to identify MDR1-related mutations and MDR1h ones, apart from ΔV/L497, *mrr1* gene was sequenced in its entirety. Because of the great diversity of *mrr1* between *B. cinerea sensu stricto* and *B. cinerea* group S, different sets of primers were designed and used for each subgroup. These primer pairs are listed in **Supplementary Table 1**.

### 2.6 Identification of *Botrytis* species/group

The collected isolates were identified as *B. cinerea sensu stricto*, *Botrytis pseudocinerea*, and *B. cinerea* group S based on diagnostic assays developed by Leroch et al. (2013) and Plesken et al. (2015). To discriminate between *B. cinerea* group S and *B. cinerea sensu stricto* or *B. pseudocinerea*, a duplex PCR was applied to detect a 21-bp insertion in *mrr1*, present only in *B. cinerea* group S using the primer pair *Mrr1*_spez_F/*Mrr1*_spez_R (Leroch et al., 2013). Similarly, by using the primer pair BcinN-in-F/BcinN-in-R, an 18-bp indel in *mrr1* gene was detected in *B. cinerea* group S or *B. pseudocinerea* isolates, but not in *B. cinerea sensu stricto* isolates (Leroch et al., 2013). The identification results of this duplex PCR assay were further verified by a second PCR assay that differentiates *B. pseudocinerea* from *B. cinerea sensu stricto* and *B. cinerea* group S, based on a *B. pseudocinerea* 24-bp deletion in the homolog of the *B. cinerea* gene BCIG_07159, using the primer pair g2944_137_F/g2944_137_R (Plesken et al., 2015). A complete list of primer pair sequences used in this study is provided in **Supplementary Table 1**. Genomic DNA was used as the template for all reactions. All PCRs were carried out in 25- μl volume with One*Taq* ^®^ 2X Master Mix with Standard Buffer (NEB) used in the following concentrations: 10 µM of Forward Primer 0.5 μl, 10 µM of Reverse Primer 0.5 μl, One*Taq* 2X Master Mix with Standard Buffer 12.5 μl, DNA 100 ng (3 μl), and nuclease-free water up to 25 μl. PCR amplification protocols were as those described by Leroch et al. (2013) and Plesken et al. (2015).

### 2.7 Determination of the synergistic effect of target site and MDR mutations on fungal sensitivity to fungicides

To test whether the coexistence of target-site mutations along with MDR ones in the same strains affects their sensitivity to fungicides, a group of 58 isolates was subjected to sensitivity measurement in terms of EC_50_ calculation to cyprodinil, boscalid, and fenhexamid. Among these 58 isolates, 24 isolates possessed only target site alternations (single, double, or triple) to *Bcpos5* (L412F), *sdhB* (H272R), and *erg27* (F412S); 26 isolates possessed both the same target site alternations, and in addition, they also possessed the ΔL/V497 deletion in *mrr1* gene associated with the MDR1h type of resistance, while eight isolates (including the reference strain B05.10) possessed neither target site nor MDR mutations and used as reference strains of wild-type sensitivity. A complete list of the isolates used for fungicide sensitivity measurements along with their phenotypic and genotypic characteristics is provided in **Supplementary Table 2**. Sensitivity measurements to boscalid were based on the inhibition of conidial germ tube growth on YBA medium amended with boscalid at concentrations of 0 μg/ml, 0.001 μg/ml, 0.005 μg/ml, 0.01 μg/ml, 0.05 μg/ml, 0.1 μg/ml, 0.5 μg/ml, 1 μg/ml, 2.5 μg/ml, 5 μg/ml, 10 μg/ml, and 15 μg/ml, following a bioassay procedure described previously (Veloukas et al., 2011). Sensitivity measurements to cyprodinil and fenhexamid were based on the inhibition of mycelial growth on Gamborg B5 or HA medium, amended with 0 μg/ml, 0.1 μg/ml, 0.05 μg/ml, 0.1 μg/ml, 0.5 μg/ml, 1 μg/ml, 5 μg/ml, 7.5 μg/ml, 10 μg/ml, 15 μg/ml, 20 μg/ml, and 30 μg/ml of cyprodinil and 0 μg/ml, 0.01 μg/ml, 0.03 μg/ml, 0.1 μg/ml, 0.3 μg/ml, 1 μg/ml, 3 μg/ml, 10 μg/ml, and 30 μg/ml of fenhexamid. The plates were incubated at 20°C in the dark for about 4 days, and the colonies’ diameter was measured after the end of the incubation period (Konstantinou et al., 2015).

### 2.8 Data analysis

The EC_50_ values were determined by plotting the relative inhibition of the diameter of mycelial growth or the relative inhibition of germ tube growth against the Log10 of fungicide concentrations. Calculations were performed with the use of SAS (JMP; SAS Institute, Cary, NC, USA). Resistance factors (RFs) were calculated by dividing the EC_50_ value of each isolate by the mean EC_50_ value of the wild-type isolates.

## 3 Results

### 3.1 Resistance frequencies to specific fungicide classes

**Figure 2:**
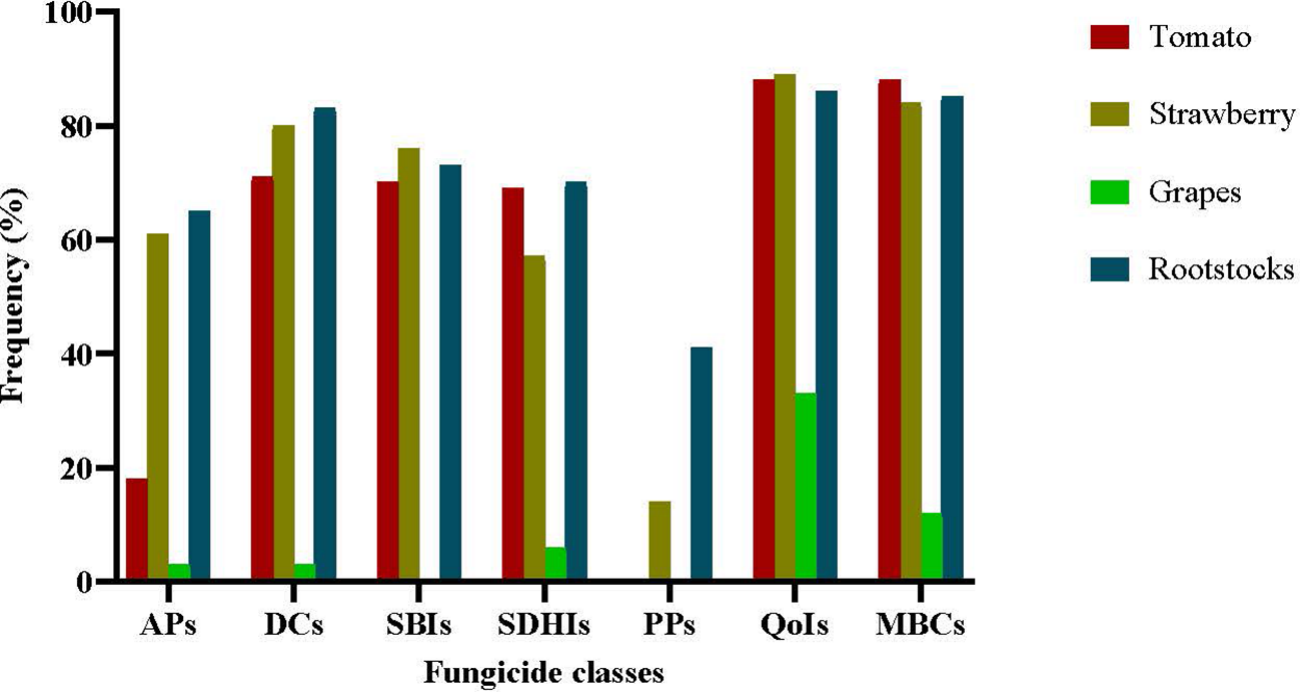
Frequencies of resistance to several fungicide classes (APs, anilinopyrimidines-cyprodinil; DCs, dicarboximides-iprodione; SBIs class III, sterol biosynthesis inhibitors class III -fenhexamid; SDHIs, succinate dehydrogenase inhibitors -boscalid and fluopyram; PPs, phenylpyrroles-fludioxonil; QoIs, quinone outside inhibitors -pyraclostrobin; MBCs, benzimidazoles-thiophanate-methyl) in *Botrytis cinerea* populations collected from several hosts.

The precise frequencies of resistance to each fungicide class in the sampled populations are provided in **Figure 2**. The *B. cinerea* population originating from grapes was the pathogen population less affected by resistance development. The entire population originating from grapes was sensitive to SBIs and PPs, while only less than 10% of the population was resistant to APs, SDHIs, and DCs. In contrast, resistance frequencies to most of the fungicides tested in the populations originating from the remaining hosts were substantially high. The higher frequencies of resistance were observed for QoIs with values of 88.6%, 89.3%, and 86.8% in the tomato, strawberry, and rootstock populations, respectively. Similarly high were the resistance frequencies to MBCs, despite the little or no use of them during the latest decades and to DCs. Resistance frequencies were lower for SBIs or SDHIs with values of 70.1%, 76.2%, and 73.7% or 69.2%, 57.1%, and 70.4% for tomatoes, strawberries, and rootstocks, respectively. While resistance frequency to APs was high in the strawberry and rootstock populations with values of 61% and 65%, respectively, only 18.2% of the isolates collected from tomato plants were resistant to this fungicide group. Isolates with resistance to PPs were found only in the populations originating from rootstock seedlings and strawberries at frequencies of 41.6% and 14%, respectively, while the populations originating from either tomatoes or grapes were completely sensitive to this fungicide class (**Figure 2**).

### 3.2 Multiple-resistance frequencies

**Figure 3:**
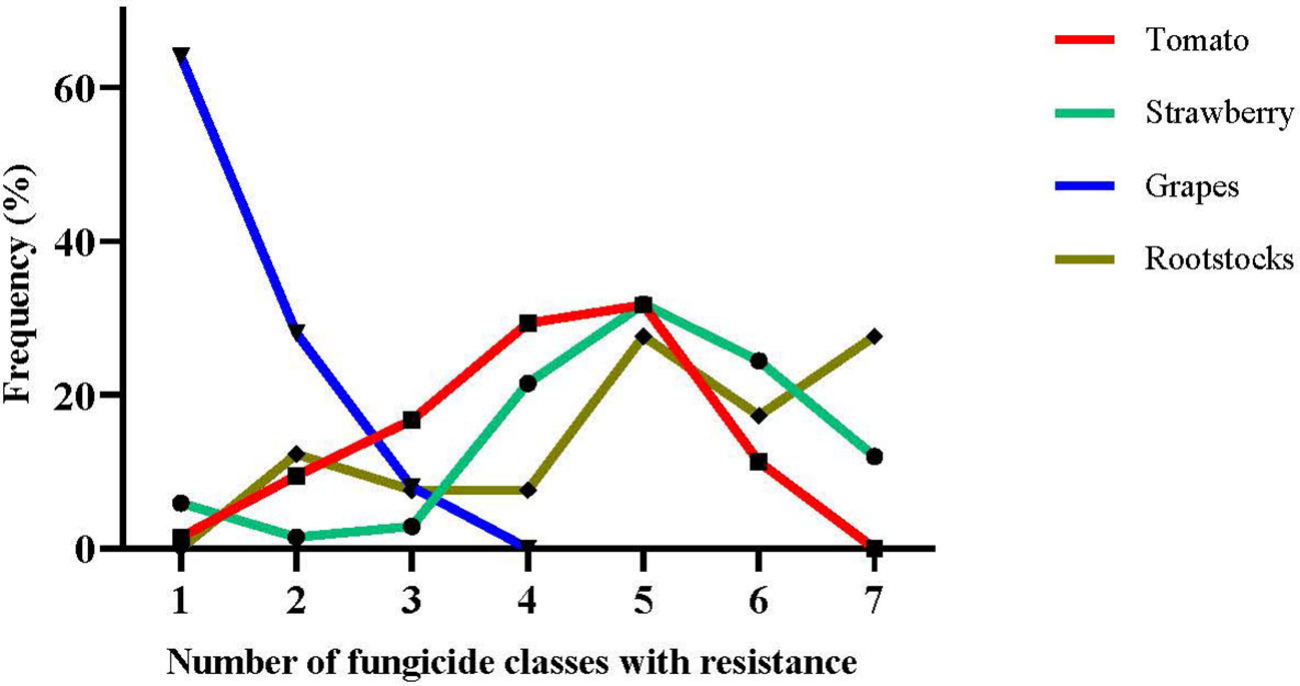
Frequencies of resistance of *Botrytis cinerea* strains originating from tomato, strawberry, grape, and stone-fruit rootstock seedling crops with multiple resistance (MLR) to several fungicide classes.

Accumulation of more than one target-site resistance-conferring mutations in the same strain can lead to MLR phenotypes. Here, we analyzed the MLR patterns of the collected isolates by categorizing them as R0 (sensitive), R1 (resistant to one fungicide class), and R2-R7 (resistant to two to seven fungicide classes simultaneously). MLR frequencies were very low in isolates originating from grapes, with most of them being sensitive to all the tested fungicides or resistant to a single fungicide class (**Figure 3**). No resistance to more than three different fungicide classes was recorded among isolates obtained from grapes. In contrast, multiple resistance phenotypes were observed in high frequencies in the populations originating from the remaining three hosts. Interestingly, the sum of R5, R6, and R7 phenotypes corresponded to more than 55% of the total population in strawberry and rootstock populations tested, while a high frequency of the isolates (27.6%) obtained from rootstock seedlings were resistant to all the seven fungicides tested (**Figure 3**).

### 3.3 Target site modifications in AP-, SBI class III-, and SDHI-resistant strains

**Figure 4:**
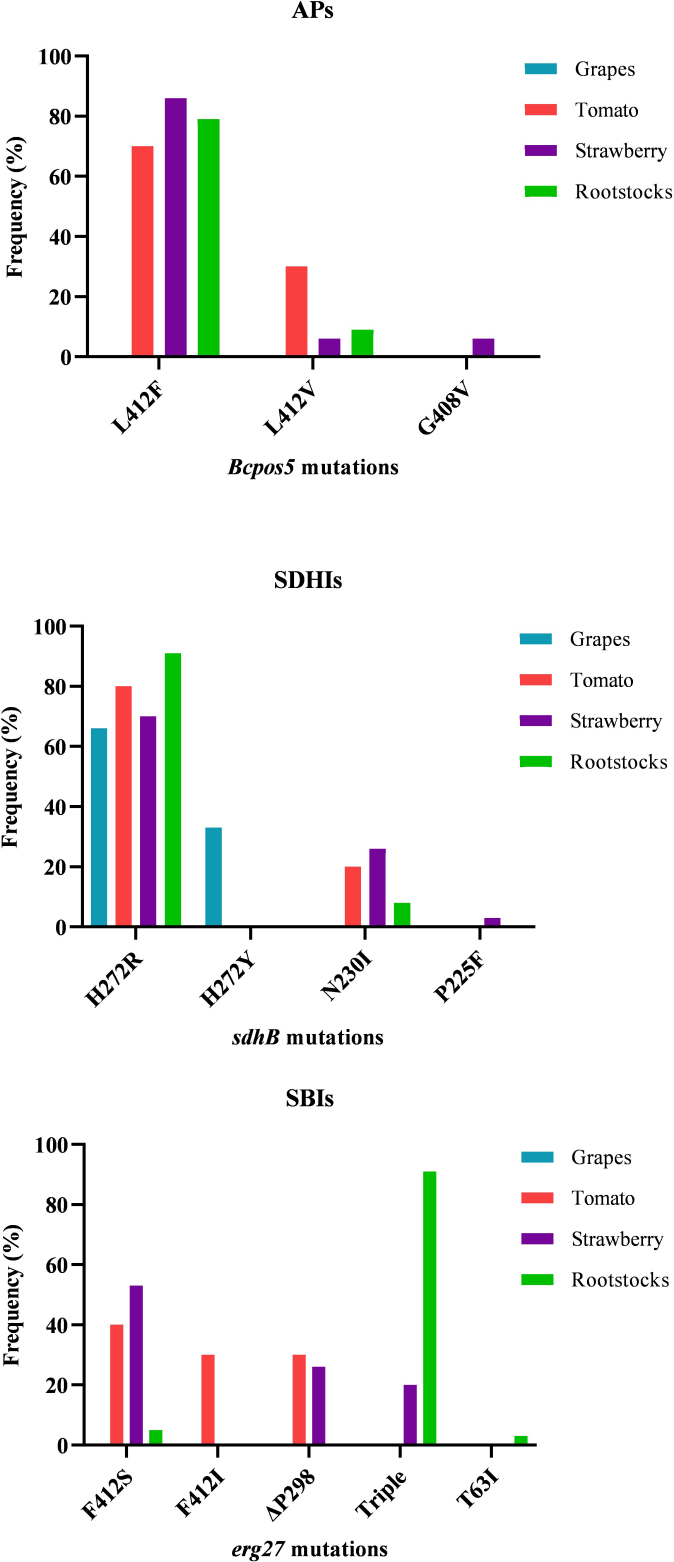
Frequencies of *Botrytis cinerea* isolates possessing different target site modifications in *BcPos5*, *sdhB*, and *erg27* genes, associated with resistance to AP, SDHI, and SBI class III fungicides, respectively. The triple mutation pattern in *erg27* consists of F412S, E263D, and P250F substitutions.

The identification of the mutations in the target genes of AP, SDHI, and SBI class III fungicides revealed a high variability of different resistance-conferring mutations. The entire spectrum and the frequencies of the identified target-site mutations in the AP-, SBI class III-, and SDHI-resistant isolates are provided in **Figure 4**. Within the AP-resistant fraction, L412F was by far the most common mutation in all the three AP-resistant subpopulations originating from strawberry, tomato, and rootstock seedlings, while L412V and G408V were found at much lower frequencies. The latter was only found in strawberry isolates. Likewise, regarding SDHIs, H272R was expectedly the dominant mutation followed by N230I, while the mutations H272Y and P225F were identified only in a very low number of isolates originating from grapes and strawberries, respectively. As for fSBIs class III, in the population originating from tomatoes, F412S was the most common *erg27* mutation in the resistant fraction of the population followed by relatively high frequencies of the F412I and ΔP298 substitutions. Similarly, in the population originating from strawberries F412S was the most common mutation followed by ΔP298 and the triple combination of F412S, E263D, and P250F mutations. In contrast, in the population originating from the rootstock seedlings, isolates possessing the triple combination of mutations (F412S, E263D, and P250F) were by far predominant in the SBI class III-resistant subpopulation with only a limited number of resistant isolates possessing either F412S or T63I mutations (**Figure 4**).

### 3.4 Phenotypic and genotypic characterization of MDR strains

Assessment of resistance to tolnaftate was used as an indicator of MDR in the isolates tested in this study. Phenotypic assessment using the discriminatory concentration of 5 μg/ml of tolnaftate revealed that 17.3% and 22.8% of the isolates originating from tomatoes and strawberries, respectively, were resistant to tolnaftate, while none of the isolates originating from grapes was found to be resistant (**Supplementary Figure 1**). In contrast, the frequency of MDR strains in the isolates set originating from rootstock seedlings reached the value of 85.4% (**Supplementary Figure 1**).

**Figure 5:**
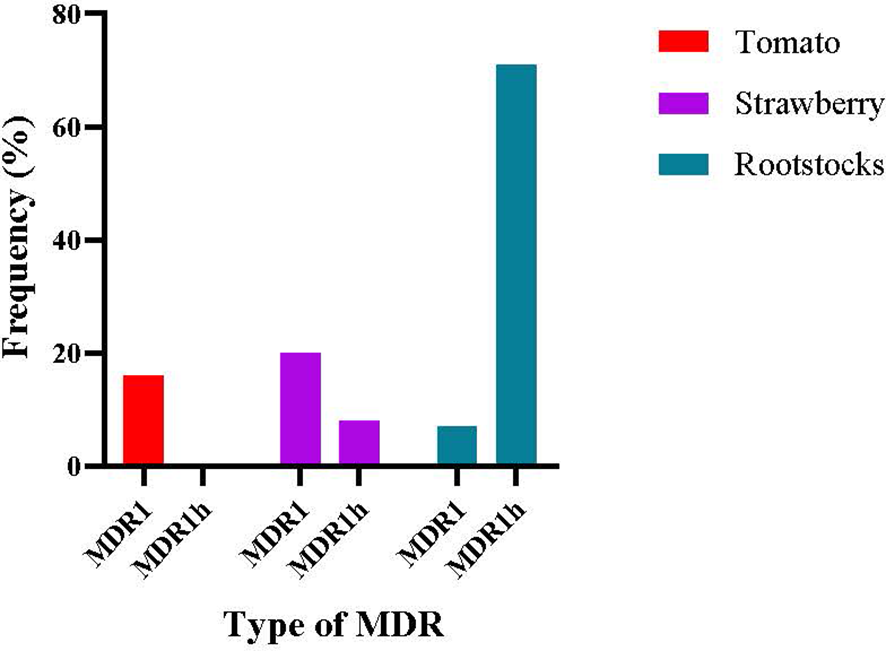
Frequency of types of multidrug resistance (MDR) in *Botrytis cinerea* isolates originating from different hosts.

The genotypic characterization of the tolnaftate-resistant isolates was conducted by using several molecular assays described in the respective Materials and Methods section. Sequencing of *mfsM2* gene showed that none of the isolates possessed the promoter rearrangement of *mfsm2*, and thus, neither MDR2 nor MDR3 genotypes were recorded from any host. The genotypic background of the tested isolates, in terms of the revealed HpyCH4V digestion profile and *mrr1* sequence data, showed that all the MDR isolates originating from tomato were classified as MDR1, accounting for a frequency of 16.1% in the total tomato population tested, while in strawberry, 19.7% and 6.2% of the isolates belonged to MDR1 and MDR1h, respectively (**Figure 5**). In contrast, the population originating from rootstock seedlings was dominated by the MDR1h genotype identified at a frequency of 73%, while only 5.9% of the population was identified as MDR1 (**Figure 5**).

**Figure 6:**
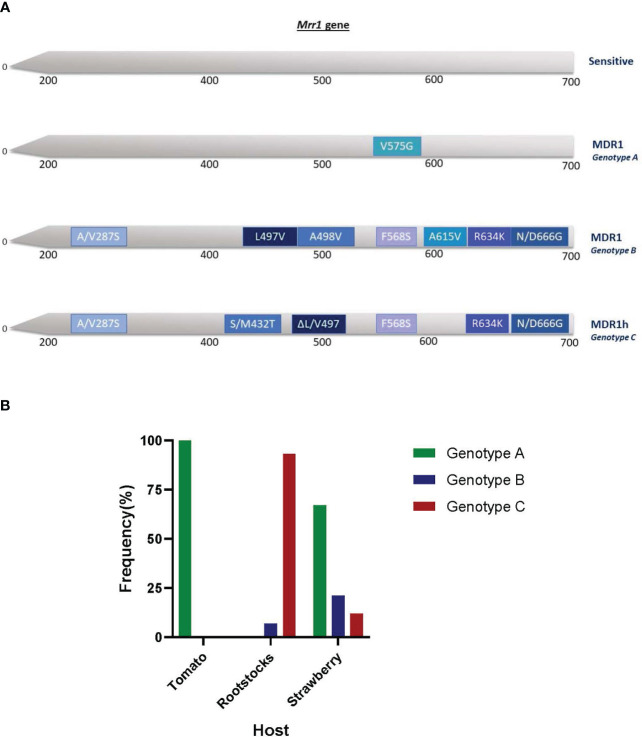
Mutations and deletions found in the transcription factor *mrr1* of multidrug-resistant strains of *Botrytis cinerea* originating from different hosts in Greece **(A)** and their respective frequencies on each host **(B)**. All mutations and deletions were indicated based on the *mrr1* sequence of the reference strain B05.10.

The sequencing of *mrr1* gene showed that three distinct genotypes existed within the MDR fraction of the tested population (**Figure 6A**). The first genotype (genotype A) was that of MDR1 isolates possessing only the V575G mutation in *mrr1* gene. This genotype was identified in all (100%) of the MDR isolates originating from tomatoes and in 67% of the MDR isolates originating from strawberries (**Figure 6B**). The second MDR1 genotype (genotype B) was characterized by a set of seven different *mrr1* mutations (A/V287S, L497V, A498V, F568S, A615V, R634K, and N/D666G) (**Figure 6A**) and was found to exist in a limited number of MDR isolates originating from rootstock seedling plants and strawberry fruit. The third genotype (Genotype C) identified by the sequencing of *mrr1* gene of the MDR isolates was that of the MDR1h strains. These strains, in addition to the ΔL/V497 deletion that characterizes the MDR1h strains, also possessed a set of five different mutations in the *mrr1* sequence (A/V287S, S/M432T, F568S, R634K, and N/D666G). Within the MDR fraction of the population, MDR1h strains were identified at a frequency of 93% in the rootstock seedling population and at a frequency of 12% in the strawberry population (**Figure 6B**).

### 3.5 Identification of *Botrytis* species/groups

**Figure 7:**
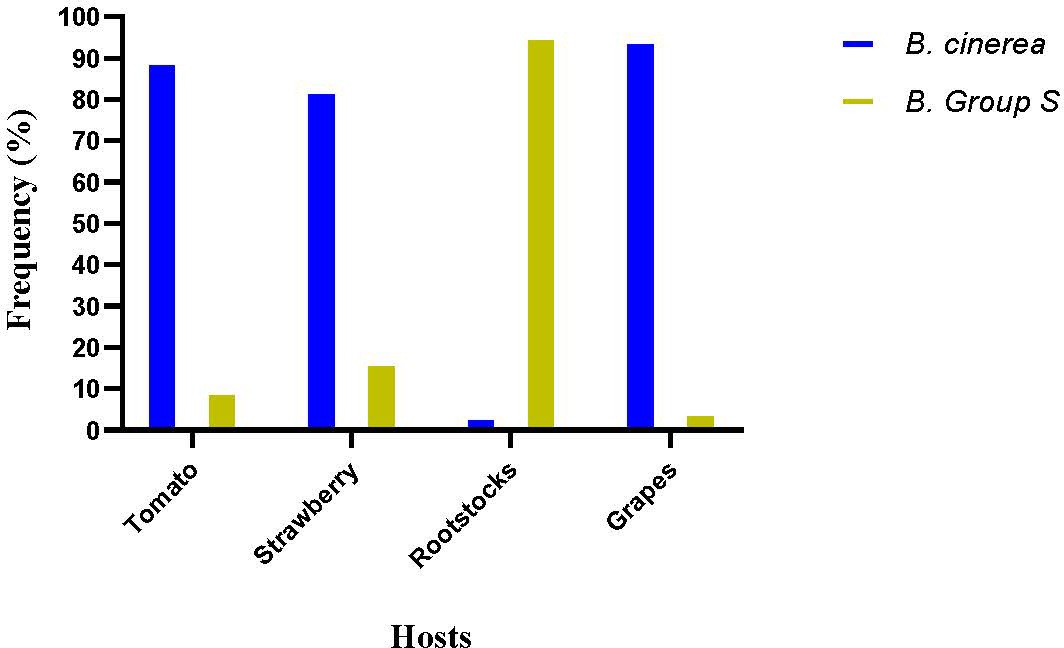
Frequencies of *Botrytis cinerea sensu stricto* and *Botrytis cinerea* group S in pathogen populations obtained from several hosts.

The molecular detection of the insertions in *mrr1* gene enabled the differentiation of the population tested in this study in *B. cinerea sensu stricto*, *B. pseudocinerea*, and *B. cinerea* group S. Results showed that there was great differentiation in the population’s composition depending on the host of origin and the intensity of selection force determined by the fungicide application history. *B. pseudocinerea* was not identified in any of the four sampled hosts. In the populations originating from tomatoes and grapes, *B. cinerea sensu stricto* was by far the predominant species, while on these hosts, *B. cinerea* group S was identified in frequencies much lower than 10% (**Figure 7**). In contrast, *B. cinerea* group S was the predominant species in rootstock seedlings, accounting for more than 90% of the total population tested. In strawberries, the frequencies of *B. cinerea* and *B. cinerea* group S accounted for 79% and 21%, respectively (**Figure 7**). In addition, all the MDR1h isolates were identified as *Botrytis* group S, whereas MDR1 isolates were identified as either *B. cinerea sensu stricto* or *B. cinerea* group S. Furthermore, all the MDR1 isolates that had been identified as *B. cinerea sensu stricto* possessed the mutation V575G in *mrr1* gene. No other mutation in *mrr1* gene was reported in MDR1 strains, identified as *B. cinerea sensu stricto*.

### 3.6 Combination of target-site and MDR mutations can lead to even greater resistance levels

**Figure 8:**
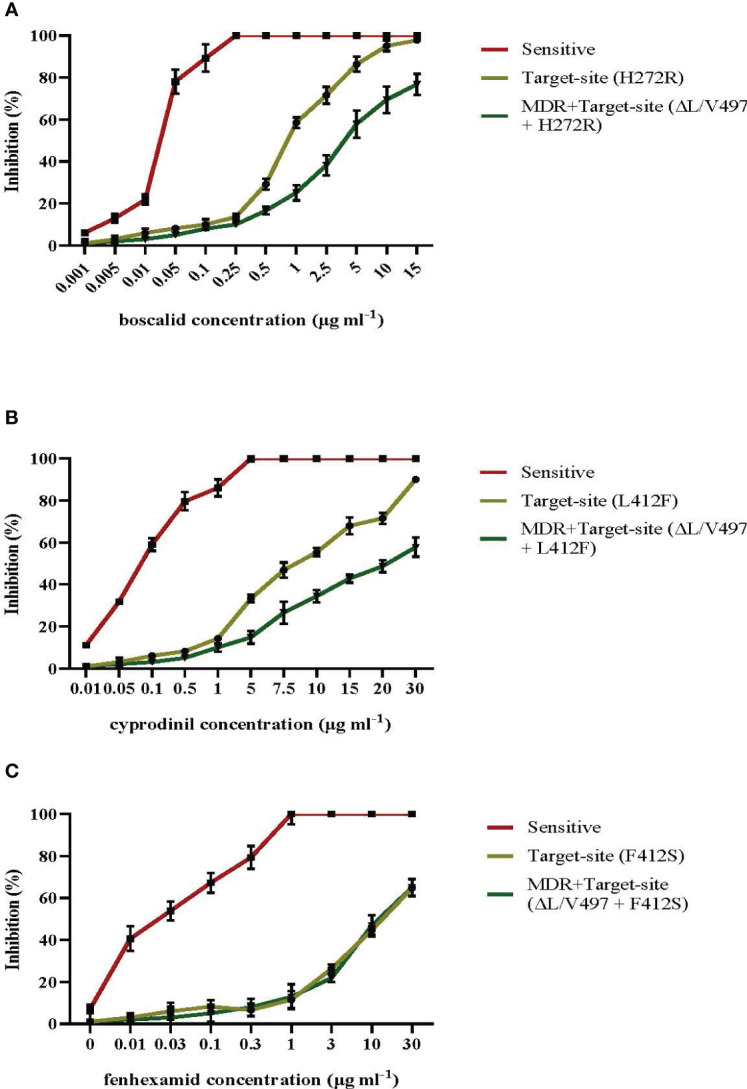
Mean relative inhibition (%) of spore germ tube elongation in the presence of different concentrations of boscalid **(A)** and mean relative inhibition (%) of mycelial growth in the presence of different concentrations of cyprodinil **(B)** or fenhexamid **(C)** of *Botrytis cinerea* isolates possessing target site alterations or both target site alterations and the *mrr1* modification ΔL/V497 leading to the MDR1h phenotype. Vertical lines indicate the standard error of the mean.

The sensitivity measurements in a wide range of pathogen isolates possessing either the most common target site modifications of F412S, H272R, and L412F in *erg27*, *sdhB*, and *Bcpos5*, respectively, or the same target-site mutations along with the MDR1h-associated modification of ΔL/V497 in *mrr1* gene showed that in isolates possessing both resistance mechanisms, there was an increased level of resistance to the AP fungicide cyprodinil and the SDHI boscalid compared to that in isolates possessing only the respective target site alterations (**Figures 8A, B**, respectively). In contrast, the inhibition of mycelial growth at the different concentrations of fenhexamid of both types of resistant isolated tested was similar (**Figure 8C**). Precise sensitivity measurements in terms of EC_50_ and the respective RF values revealed that isolates possessing only target site modifications in *sdhB* or *BcPos5* showed RF values ranging from 32.5 to 72.5 and from 27.5 to 45.5 for boscalid and cyprodinil, respectively, while isolates possessing both target site alterations and the MDR1h mechanism of resistance showed higher RF values ranging from 24.5 to 170 and from 37.4 to >120 for boscalid and cyprodinil, respectively (**Supplementary Table 2**). However, no marked differences in the levels of resistance to fenhexamid were observed among isolates possessing only the F412S mutation in *erg27* or both the F412S and MDR1h deletions with RF values ranging from 90.4 to 371 and from 78 to 371, respectively (**Supplementary Table 2**).

## 4 Discussion

Monitoring data obtained in this study showed a widespread presence of resistance to several fungicide classes in *B. cinerea* populations in the most heavily treated crops in Greece. Such data confirm previous reports suggesting the development of high resistance frequencies in fungal populations originating from several hosts to APs, QoIs, SDHIs, DCs, or SBIs class III in several regions of Greece (Myresiotis et al., 2007; Avenot et al., 2008; Bardas et al., 2010; Samuel et al., 2011; Konstantinou et al., 2015). The resistance frequencies in fungal populations originating from strawberry, greenhouse- grown tomato, or rootstock seedlings were high for almost all fungicide classes, with the exception of PPs. All these crops are grown in closed environments such as greenhouses or high height tunnels that facilitate the development of high relative humidity conditions favoring the development of the disease and necessitating a high number of fungicide spray applications to successfully combat it. In contrast, lower resistance frequencies were observed in the populations originating from wine grapes. The dry climate of the Mediterranean region contributes to a relatively low risk for gray mold development on grapes and, thus, in a limited number of botryticide applications in contrast to northwestern Europe where grapevines are heavily treated with botryticides. Similarly low resistance frequencies have been observed in *B. cinerea* populations from grapes in Australia, while in northwestern European countries, resistance frequencies in the grape populations are higher (Leroch et al., 2011; Walker et al., 2013; Harper et al., 2022). Differences in the frequencies of fungicide resistance within *B. cinerea* populations originating from different hosts have been observed on several crops and several places of the world and have been correlated with differences in the level of crop susceptibility to the pathogen, the total number of fungicide spray applications applied on the crop, and the availability of cultural control methods as an alternative to chemicals (Cosseboom and Hu, 2021.)

In addition to the observed high resistance frequencies to specific fungicide classes in heavily treated crops, the results of this study showed that in the same crops, high frequencies of isolates showing resistance to multiple modes of action (MLR) have been selected. The observed frequencies of MLR strains in the populations originating from strawberries and tomatoes were higher compared to the respective frequencies reported in a previous survey from the same region (Konstantinou et al., 2015). Thus, in the current study, we documented a further increase in MLR frequencies within the fungal population. Multiple resistance to fungicides in *B. cinerea* is a rapidly expanding problem in the fungal populations worldwide, reported in several studies during the last decade (Leroch et al., 2011; de Miccolis Angelini et al., 2014; Fernandez-Ortuno et al., 2014; Li et al., 2014; Lu et al., 2016; Campia et al., 2017; Rupp et al., 2017; Cosseboom et al., 2020; Makris et al., 2022; Naegele et al., 2022). The observed increased frequencies of MLR strains in populations from crops heavily treated with fungicides are obviously associated with the high number of sprays required to successfully control the disease, the application of pre-packed mixtures of different active ingredients that are of high risk for resistance development, or the absence of effective alternative control methods that could contribute to a reduction of selection pressure. Moreover, in addition to botryticides applied against gray mold, the fungal population on some of these hosts may be exposed to fungicides that are applied against other foliar or fruit diseases. Such an exposure increases the selection pressure on the fungal population and may contribute to the increased MLR frequencies observed in our study.

Sequence analysis of the target genes of boscalid (SDHI), cyprodinil (AP), and fenhexamid (SBIs class III) uncovered a variety of mutations associated with target-site resistance. In the boscalid-resistant fraction of the populations originating from all the sampled hosts, H272R was found to be, by far, the most common mutation followed by N230I, while other mutations such as H272Y or P225F were found in very low frequencies. These results are in line with the results of several other studies conducted worldwide, suggesting that H272R is the most common *sdh*B mutation conferring resistance to some of the SDHI fungicides (Hu et al., 2016; Harper et al., 2022). Interestingly, in our study, an increase of the N230I mutation frequency accompanied by a decrease in the frequency of H272Y mutation was observed, compared to frequencies reported in previous monitoring studies conducted in Greece on the same hosts and the same sampling regions (Veloukas et al, 2011; Konstantinou et al., 2015). This type of change in the frequencies of these mutations is most probably associated with the introduction into the spray programs of a new SDHI fungicide, fluopyram, which has been registered for use on tomatoes, strawberries, or grapes against several diseases (Veloukas and Karaoglanidis, 2012). It is well established that N230I confers resistance to several SDHIs including fluopyram, while H272Y is associated with hypersensitivity to fluopyram (Veloukas et al., 2013; Laleve et al., 2014; Fernandez-Ortuno et al., 2017). Thus, the introduction of fluopyram into the spray programs led to a reduction of the fluopyram-hypersensitive H272Y strains and favored the selection of fluopyram-resistant N230I strains. Overall, these data confirm the necessity of continuous monitoring for changes in the *sdh* gene mutation frequencies within the fungal populations to adopt the appropriate resistance management strategies since it is well established that the different *sdh* mutations affect in different ways the level of sensitivity to various SDHIs and the fitness of the resistant strains (Veloukas et al., 2013; Laleve et al., 2014; Veloukas et al., 2014).

Sequencing of *erg27* gene in the fenhexamid-resistant fraction of the population revealed the presence of a wide array of mutations associated with resistance to SBI class III fungicides. In total, within the fenhexamid-resistant fraction of the population, six different mutations were identified, with F412S being predominant in the populations originating from tomatoes and strawberries. In contrast in the population originating from stone-fruit rootstock seedlings, the predominant genotype was that of isolates possessing a combination of three different *erg27* mutations (F412S, E263D, and P250F). All these six mutations have been reported previously in several monitoring studies (Grabke et al., 2013; Avenot et al., 2020; Alzohairy et al., 2021). Mutations located in codon 412 of *erg27* strongly affect the binding of SBI class III fungicides to their target site and are associated with high resistance levels to fenhexamid. F412S has been reported as the most common *erg27* mutation conferring resistance to this fungicide class, wherever resistance to SBIs class III has been developed; thus, our results are in agreement with these reports (Fillinger et al., 2008; Grabke et al., 2013).

Resistance to APs is also widespread throughout the world (Latorre et al., 2002; Leroch et al., 2013; Shao et al., 2021), while in Greece, the first cases of APs resistance in *B. cinerea* populations were documented in 2007 (Myresiotis et al., 2007). Despite the fact that APs is a relatively old fungicide class introduced against gray mold during the 1990s and the first cases of resistance were reported in the late 1990s (Chapeland et al., 1999), it was only in 2017 when Mosbach et al. (2017) identified several AP resistance-conferring mutations in genes encoding proteins that are involved in mitochondrial processes, providing evidence that APs are primarily targeting the mitochondrial metabolism. Among several mutations identified as AP resistance-conferring mutations, L412F in *Bcpos5* and *E407K* in *Bcmdl1* were included. The former has been found as the predominant mutation in the AP-resistant subpopulation tested by Mosbach et al. (2017), while, more recently in an AP-resistant subpopulation originating from China, the latter was found to be predominant (Fan et al., 2023).

In addition to target site resistances in the fungal population, the presence of isolates showing MDR phenotype was evident. These isolates were showing low-to- moderate levels of resistance to fludioxonil, a fungicide for which only rarely has been reported target site resistance in *B. cinerea* because of the low fitness of the resistant isolates (Dowling et al., 2021; Wang et al., 2021). The sequencing of the transcription factor gene *mrr1* in these isolates confirmed their identity as MDR strains. The presence of MDR strains is confirmed for the first time in Greece, while previously their presence has been reported in Germany, France, the USA, and China (Kretschmer and Hahn, 2008; Kretschmer et al., 2009; Leroch et al., 2013; Fernandez-Ortuno et al., 2014; Shao et al., 2021). The sequencing of *mrr1* gene of the MDR strains revealed the presence of three distinct genotypes. The predominant genotype was that of the MDR1h strains characterized by the 3-bp deletion at codon 497 in the *mrr1* exon. Apart from this deletion, the MDR1h isolates had several target point alterations throughout *mrr1* gene. MDR1h was identified for the first time in strawberry fields in Germany (Fournier et al., 2005; Leroch et al., 2013), while, later on, it was also reported in strawberry fields in the USA (Fernandez-Ortuno et al., 2015). The expression levels of *atrB* in MDR1h strains are generally higher compared to those of MDR1 leading consequently to higher levels of resistance to fungicides (Leroch et al., 2013; Fernandez-Ortuno et al., 2015). MDR1h strains were found to be the predominant MDR strains in the population originating from rootstock seedlings, while they were completely absent in the population originating from tomatoes. The predominance of MDR1h strains on rootstock seedlings is associated with the fact that this crop is the most heavily treated crop with fungicides among those sampled, while it is well established that the selection of MDR1h strains is stronger in most heavily treated crops than in crops where fungicide use is less common (Leroch et al., 2013). The second MDR genotype identified in the current study was a set of MDR1 isolates possessing seven different mutations in the *mrr1* sequence. Most of these mutations have already been reported as *mrr1* mutations associated with the overexpression of *BcatrB* in MDR1 isolates from Europe, Australia, and the USA (Kretschmer et al., 2009; Leroch et al., 2013; Fernandez-Ortuno et al, 2015; Harper et al., 2022). Interestingly, the third genotype identified as MDR1 was a set of isolates that possessed only the V575G substitution in *mrr1* gene. All the MDR1 isolates originating from tomatoes possessed this mutation, while it was also observed in a limited number of isolates originating from strawberries. This mutation is reported for the first time; however, another mutation in the same codon (V575M) has been previously reported by Leroch et al. (2013) and was associated with the overexpression of *BcatrB*. All the isolates possessing this mutation have been identified as *B. cinerea sensu stricto*.

Interestingly, in all of our MDR1 or MDR1h isolates, the resistance mechanism associated with *atrB* overexpression coexisted with target site modifications in the same strains. The higher accumulation of target site resistances in MDR strains of the pathogen has been observed in the past in *B. cinerea* populations from several hosts in Germany (Leroch et al., 2013) and from strawberries in the USA and Norway (Fernandez-Ortuno et al., 2015; Nielsen et al., 2022). Thus, our results provide further evidence supporting the theory of a stepwise pattern of accumulation of resistance mutations in *B. cinerea* populations (Li et al., 2014). However, little is known about the effect of the coexistence of different resistance mechanisms on the levels of resistance of *B. cinerea* strains. In general, overexpression of efflux transporters as a resistance mechanism leads to low/moderate levels of resistance in plant pathogenic fungi (Leroch et al., 2013). The combination of different resistance mechanisms in the same strain may lead to increased resistance levels as has been shown in fungal species such as *P. digitatum* or *Z. tritici* (Omrane et al., 2015; de Ramon-Carbonell and Sanchez-Torres, 2020) Herein, we provided evidence that the coexistence of two different resistance mechanisms increases the level of resistance to some fungicides and thus increases the risk for the lower performance of the fungicides that are affected. However, this was not the case for fenhexamid for which the coexistence of *erg27* mutations with *mrr1* modifications did not lead to resistance levels higher than those obtained due to the *erg27* modifications. This result is in accordance with previous studies showing that overexpression of *BcatrB* (and thus MDR1/MDR1h) is not correlated with increased detoxification of fenhexamid (Kretschmer et al., 2009).

In conclusion, in the current study, we reported on changes in the multiple resistance frequencies of *B. cinerea* populations originating from several hosts in Greece and on the frequencies of multidrug-resistant strains, which are reported for the first time in Greece. The data presented in this study highlight the practical importance of MLR/MDR in the field since resistance levels against certain fungicides might be higher than previously anticipated and might be used as a tool to design resistance management programs. The observed high frequencies of MLR isolates and the confirmed presence of MDR strains within the fungal populations present a challenge for the control of the pathogen, particularly in crops where multiple botryticide applications are required. This challenge necessitates research toward the development of fungicides with novel modes of action or the adoption of novel control strategies that will lead to the successful control of the selected MLR and MDR strains and the reduction of the selection pressure.

## Data availability statement

The raw data supporting the conclusions of this article will be made available by the authors, without undue reservation.

## Author contributions

GS: Data curation, Investigation, Writing – original draft. AS: Data curation, Investigation, Writing – original draft. GK: Funding acquisition, Supervision, Writing – review & editing.
