# Supplementary figures and images for: Multiple and multidrug resistance in Botrytis cinerea: molecular mechanisms of MLR/MDR strains in Greece and effects of co-existence of different resistance mechanisms on fungicide sensitivity
Source: Front Plant Sci. 2023 Oct 5;14:1273193. doi: 10.3389/fpls.2023.1273193 (PMC10585064; doi:10.3389/fpls.2023.1273193)

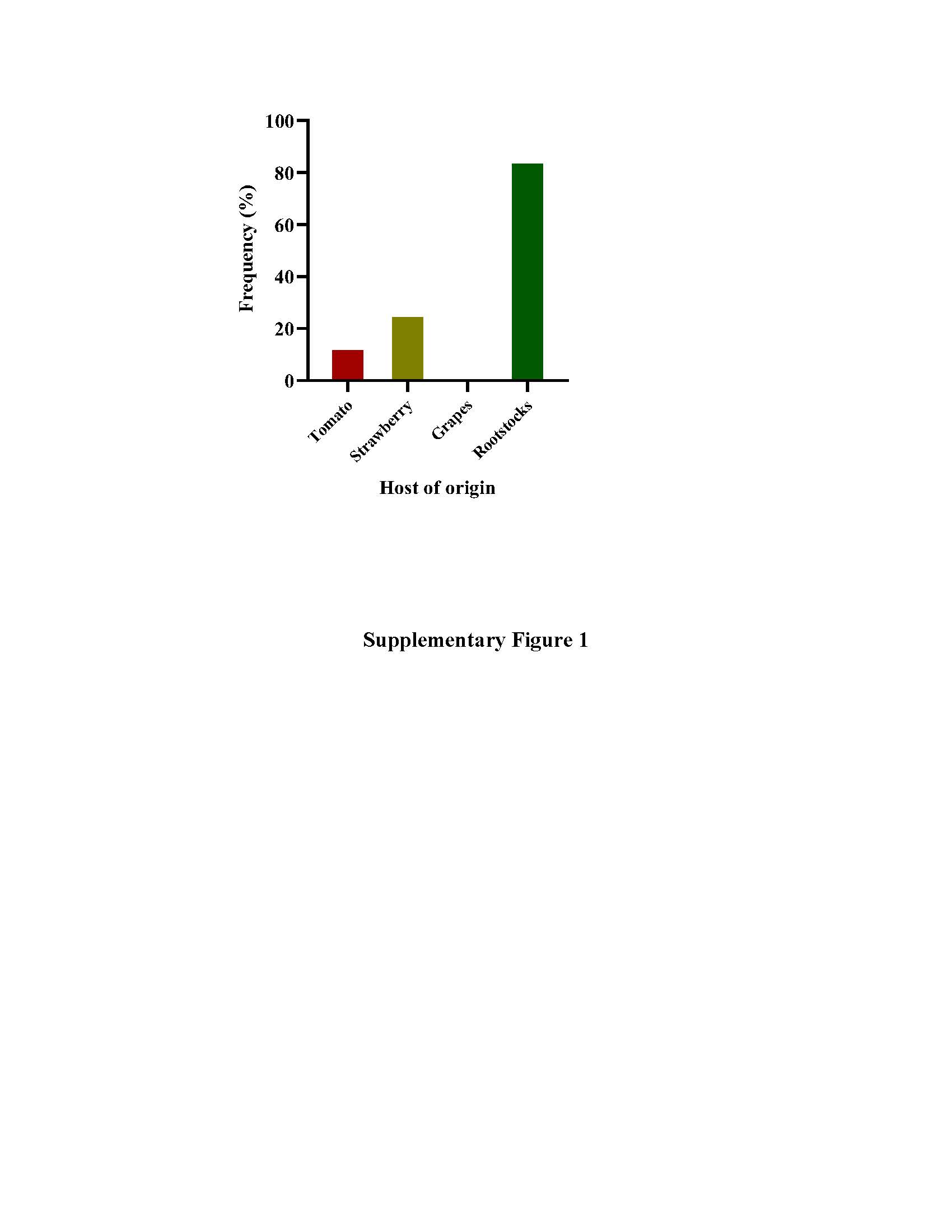

Supplement: Supplementary file 1 [file Image_1.jpeg]
