# Supplementary material for: Multiple and multidrug resistance in Botrytis cinerea: molecular mechanisms of MLR/MDR strains in Greece and effects of co-existence of different resistance mechanisms on fungicide sensitivity
Source: Front Plant Sci. 2023 Oct 5;14:1273193. doi: 10.3389/fpls.2023.1273193 (PMC10585064; doi:10.3389/fpls.2023.1273193)
Supplement: Supplementary file 2 [file Table_1.docx]

Supplementary Table 1. List of primer sequences used for target gene amplification in PCR assays

| Primer name | sequence (5’-3’) | product length (bp) | target gene | Reference |
| --- | --- | --- | --- | --- |
| Mrr1_spez_F | TATCGGTCTTGCAGTCCGC | +21bp indel | *Mrr1* | Leroch et al., 2013 |
| Mrr1_spez_R | TTCCGTACCCCGATCTTCGGAA |  |  |  |
| BcinN-in-F | GCGACCTCATCGTTCTTTCAC | +18bp indel | *Mrr1* | Plesken et al., 2015 |
| BcinN-in-R | GGCTCTCGATGAGCTGTTTC |  |  |  |
| g2944_137_F | GCAGATGAGGCGGATGATAG | +24bp indel | *BC1G_07159* |  |
| g2944_273_R | TCCACCCAAGCATCATCTTC |  |  |  |
| IpBcBeg | CCACTCCTCCATAATGGCTGCTCTCCGC | 953 | *sdhB* | Leroux et al., 2010 |
| IpBcEnd | CTCATCAAGCCCCCTCATTGATATC |  |  |  |
| H272R-fw | GGCAGCTTTGGATAACAGCATGAGTTTGTACAGATGGC | 120 | *sdhB* | Veloukas et al., 2011 |
| H272-rev | GCCATTTCCTTCTTAATCTCCGC |  |  |  |
| sdhC1  sdhC2  sdhC3  sdhC4  sdhC5 | ATCACGTGCCAGATTTCCTT | sequencing | *SdhC* | Leroux et al., 2010 |
|  | TTACAAGCCCTCCAAATTGC |  |  |  |
|  | CCAAATAAAGGCGAAGCAAG |  |  |  |
|  | TGCCTCCCAAGACAGATACC |  |  |  |
|  | AGGTGGGATCTTGGGGTTAT |  |  |  |
| sdhD1 | TGATGACCTTGCACGTGACT | sequencing | *sdhD* |  |
| sdhD2 | AGCAGCATGTGTTTGACAGC |  |  |  |
| erg27Beg | TGGGATTACCACCATGGGAGACAAGTG | 1528 | *erg27* | Fillinger et al., 2008 |
| erg27End | CAATGGTTCCGCATTTCTTTGCCTCCC |  |  |  |
| BcposF | TCGCCACCTCGAAACCTTTT | 1398 | *Bcpos5* | This study |
| BcposR2 | TCCACTCACAAAATAGCAGGGT |  |  |  |
| BcposF2 | CTAGGGTTCTTGGGCGAGTG | sequencing | *Bcpos5* | This study |
| BcposR | CATAACACAAGGAACGCCCC |  |  |  |
| mfsM2-pfor | TAGCCAATGGATCCTACG | 1625bp for sensitive, 2273bp for MDR2/3 | *MfsM2* | Kretschmer M. et al, 2009 |
| mfsM2-prev | CGAGATGGATGCCATTTCAGAG |  |  |  |
| Mrr1fora | CCATCCCACGACCATACCAA | 555 | *Mrr1*  *(B. cinerea)* | This study |
| Mrr1reva | GGGATCAGAACCTGCGAGAT |  |  |  |
| Mrr1-3rdf | TGCTTCCTCGAGGACAATCC | 700 |  |  |
| Mrr1-3rdr | TCTGTTCTTACATGCTCCCCA |  |  |  |
| Mrr ext f | TCAGCATCAGCACCGAAAGT | 2300 | *Mrr1*  *(B. group S)* | This study |
| Mrr ext r | TTTTGGGGTCCATCGCTACC |  |  |  |
| Mrr int f | TATGCGCGCTCATGATCGAA | sequencing |  |  |
| Mrr int r | TCTCCTTCGCATTTCCAAATCG |  |  |  |
