# Supplementary material for: Multiple and multidrug resistance in Botrytis cinerea: molecular mechanisms of MLR/MDR strains in Greece and effects of co-existence of different resistance mechanisms on fungicide sensitivity
Source: Front Plant Sci. 2023 Oct 5;14:1273193. doi: 10.3389/fpls.2023.1273193 (PMC10585064; doi:10.3389/fpls.2023.1273193)
Supplement: Supplementary file 3 [file Table_2.docx]

**Supplementary Table 2.** List of *Botrytis cinerea* isolates possessing either target site specific or both target-site specific and MDR mechanisms of resistance used for sensitivity measurements to the fungicides boscalid, cyprodinil and fenhexamid along with their phenotypic and genotypic background.

| **Isolate** | **Host** | **Resistance mechanism** | | **EC_50_ (μg ml^-1^)** | | |
| --- | --- | --- | --- | --- | --- | --- |
|  |  | **Target Site alteration^a^** | **MDR^b^** | **boscalid** | **cyprodinil** | **fenhexamid** |
| Α25 | tomato | APs/SDHIs/HAs | - | 1.7 (42.5) | 8.2 (32.2) | 9.85 (142.7) |
| Α62 | tomato | APs/SDHIs/HAs | - | 2.1 (52.0) | 7.9 (31.1) | 18.5 (268.1) |
| Β13 | tomato | APs/SDHIS/HAs | - | 2.2 (55.0) | 7.6 (29.9) | 15.6 (226) |
| Β36 | tomato | APs/SDHIs | - | 1.3 (32.5) | 8.7 (34.8) | 0.07 (1.0) |
| Β37 | tomato | APs/SDHIs | - | 1.7 (42.5) | 8.5 (33.4) | 0.045 (0.6) |
| Β55 | strawberry | SDHIS/HAs | - | 1.75 (43.7) | 0.36 (1.4) | 6.33 (90.4) |
| Β65 | strawberry | APs/SDHIs | - | 2.2 (55.0) | 7.0 (27.5) | 0.048 (0.7) |
| Β67 | strawberry | APs | - | 0.07 (1.7) | 7.2 (28.2) | 0.031 (0.5) |
| Β70 | strawberry | SDHIs | - | 1.6 (42.1) | 0.48 (1.9) | 0.080 (1.1) |
| Β76 | strawberry | APs/SDHIs | - | 1.85 (46.2) | 7.8 (30.7) | 0.065 (0.9) |
| Β81 | strawberry | APs/SDHIs/HAs | - | 2,3 (57,5) | 8.54 (33.6) | 8.4 (121.7) |
| Β85 | strawberry | SDHIS/HAs | - | 1.46 (36.5) | 0.32 (1.3) | 7.8 (113.4) |
| Β86 | strawberry | APs/SDHIs/HAs | - | 2.2 (55.0) | 7.8 (30.7) | 15.1 (218.8) |
| Β87 | strawberry | SDHIs | - | 2.9 (72.5) | 0.29 (1.1) | 0.045 (0.6) |
| Β89 | strawberry | APs/SDHIS/HAs | - | 1.38 (34.5) | 8.5 (33.4) | 11.2 (162.3) |
| Β90 | strawberry | APs | - | 0.06 (1.5) | 8.2 (34.1) | 0.065 (0.9) |
| Β91 | strawberry | APs/SDHIS/HAs | - | 2.65 (66.2) | 8.8 (34.6) | 15.6 (226.0) |
| Β94 | strawberry | APs/SDHIs | - | 1.4 (35) | 11.56 (45.5) | 0.035 (0.5) |
| Β98 | strawberry | APs/SDHIs/HAs | + | 4.7 (117.5) | >30 (>120) | 10.6 (151.4) |
| Β99 | strawberry | APs/SDHIS/HAs | - | 1.73 (43.2) | 7.8 (31.2) | 18.6 (269.5) |
| Β118 | strawberry | APs/SDHIs | - | 1.65 (41.2) | 7.2 (28.3) | 0.037 (0.5) |
| C3 | strawberry | SDHIs | + | 2.5 (62.5) | 2.5 (10.0) | 0.085 (1.2) |
| C10 | strawberry | APs/SDHIs/HAs | - | 2.8 (70) | 10.63 (41.8) | 21.1 (301) |
| C18 | strawberry | APs/SDHIs/HAs | + | 5.1 (127.5) | 9.5 (37.4) | 18.7 (287.6) |
| C62 | strawberry | HAs | - | 0.05 (1.1) | 0.4 (1.6) | 9.5 (135) |
| C63 | strawberry | APs/SDHIs/HAs | - | 1.4 (35) | 11.63 (45.7) | 14.8 (214.5) |
| C78 | strawberry | SDHIS/HAs | - | 2.1 (52.0) | 0.37 (1.5) | 26 (371) |
| C79 | strawberry | APs/SDHIs | + | 0.98 (24.5) | 7.8 (30.7) | 0.075 (1.1) |
| Ap1 | Rootstocks | APs/SDHIs/HAs | + | 4.3 (107.5) | 17 (66.9) | 7.5 (107) |
| Ap8 | Rootstocks | SDHIs | + | 4.8 (120) | 2.1 (8.4) | 0.060 (0.9) |
| Ap10 | Rootstocks | APs/SDHIs/HAs | + | 5.75 (143.7) | 21.4 (85.6) | 9.3 (134.8) |
| Ap11 | Rootstocks | APs/SDHIs | + | 4.5 (112.5) | >30 (>120) | 7.17 (102.4) |
| Ap12 | Rootstocks | APs/SDHIs/HAs | + | 4.01 (100.2) | 30 (120) | 22.3 (312) |
| Ap14 | Rootstocks | APs/SDHIs | + | 4.8 (120) | 14 (55.1) | 0.045 (0.6) |
| Ap15 | Rootstocks | APs/SDHIs/HAs | + | 5.2 (130) | 12.5 (49.2) | 23.2 (336.2) |
| Ap16 | Rootstocks | APs/SDHIs/HAs | + | 4.9 (122.5) | >30 (>120) | 17.6 (251.4) |
| Ap17 | Rootstocks | APs/SDHIs/HAs | + | 5.5 (137.5) | 12 (43.3) | 5 (78) |
| Ap19 | Rootstocks | APs/SDHIs/HAs | + | 4.6 (115) | 18.7 (74.8) | 11.17 (159) |
| Ap20 | Rootstocks | APs/SDHIs | + | 4.22 (105.5) | >30 (>120) | 0.085 (1.2) |
| Ap21 | Rootstocks | APs/SDHIs/HAs | + | 4.9 (122.5) | 25.2 (100.8) | 18.5 (264.2) |
| Ap23 | Rootstocks | APs/SDHIs | + | 5.1 (127.5) | >30 (>120) | 0.048 (0.7) |
| Ap30 | Rootstocks | APs/SDHIs/HAs | + | 4.6 (115) | 14.5 (57.09) | 21.2 (307.2) |
| Ap31 | Rootstocks | APs/SDHIs/HAs | + | 4.2 (105) | >30 (>120) | 9.67 (138.1) |
| Ap32 | Rootstocks | SDHIs/HAs | + | 4.87 (121.7) | 2.8 (11.2) | 17.8 (258.0) |
| Ap35 | Rootstocks | APs/SDHIs/HAs | + | 3.06 (76.5) | 25.0 (100) | 9.1 (131.9) |
| Ap36 | Rootstocks | SDHIs/HAs | + | 4.0 (100) | 3.1 (12.4) | 26 (371.4) |
| Ap37 | Rootstocks | APs/SDHIs/HAs | + | 4.81 (120.2) | 27.83 (109.5) | 15.6 (226.0) |
| Ap38 | Rootstocks | SDHIs | + | 6.8 (170) | 2.2 (8.8) | 0.075 (1.1) |
| Ap40 | Rootstocks | APs/SDHIs/HAs | + | 4.9 (122.5) | >30 (>120) | 18.5 (268.1) |
| Ap45 | Rootstocks | SDHIs | + | 4.01 (100.2) | 2.5 (10) | 0.052 (0.7) |
| C1 | Grapes | - | - | 0.085 (2.0) | 0.34 (1.3) | 0.048 (0.7) |
| C8 | Grapes | - | - | 0.06 (1.0) | 0.5 (2.0) | 0.023 (0.3) |
| C9 | Grapes | - | - | 0.08 (2.0) | 0.45 (1.8) | 0.018 (0.3) |
| C10 | Grapes | - | - | 0.08 (2.0) | 0.43 (1.7) | 0.017 (0.3) |
| BS3 | Tomato | - | - | 0.056 (1.4) | 0.37 (1.5) | 0.033 (0.5) |
| BS8 | Tomato | - | - | 0.034 (0.9) | 0.4 (1.6) | 0.021 (0.3) |
| G1 | Grapes | - | - | 0.055 (1.3) | 0.34 (1.3) | 0.037 (0.5) |
| B05.10 | Lab | - | - | 0.04 (1.0) | 0.25 (1) | 0.069 (1.0) |

^a^ isolates resistant to anilinopyrimidines (APs) were possessing the L412F mutation in *BcPos5*, to succinate dehydrogenase inhibitors (SDHIs) the H272R mutation in *sdhB* and to hydroxyanilides (HAs) the F412S mutation in *erg27*

^b^ all the MDR isolates were of the MDR1h type

^c^ Numbers in parenthesis are the Resistance Factor values and they were calculated by dividing the EC_50_ of each resistant isolate in each fungicide with the respective EC_50_ value of the reference sensitive isolate B05.10.
